# Supplementary figures and images for: Anvi’o: an advanced analysis and visualization platform for ‘omics data
Source: PeerJ. 2015 Oct 8;3:e1319. doi: 10.7717/peerj.1319 (PMC4614810; doi:10.7717/peerj.1319)

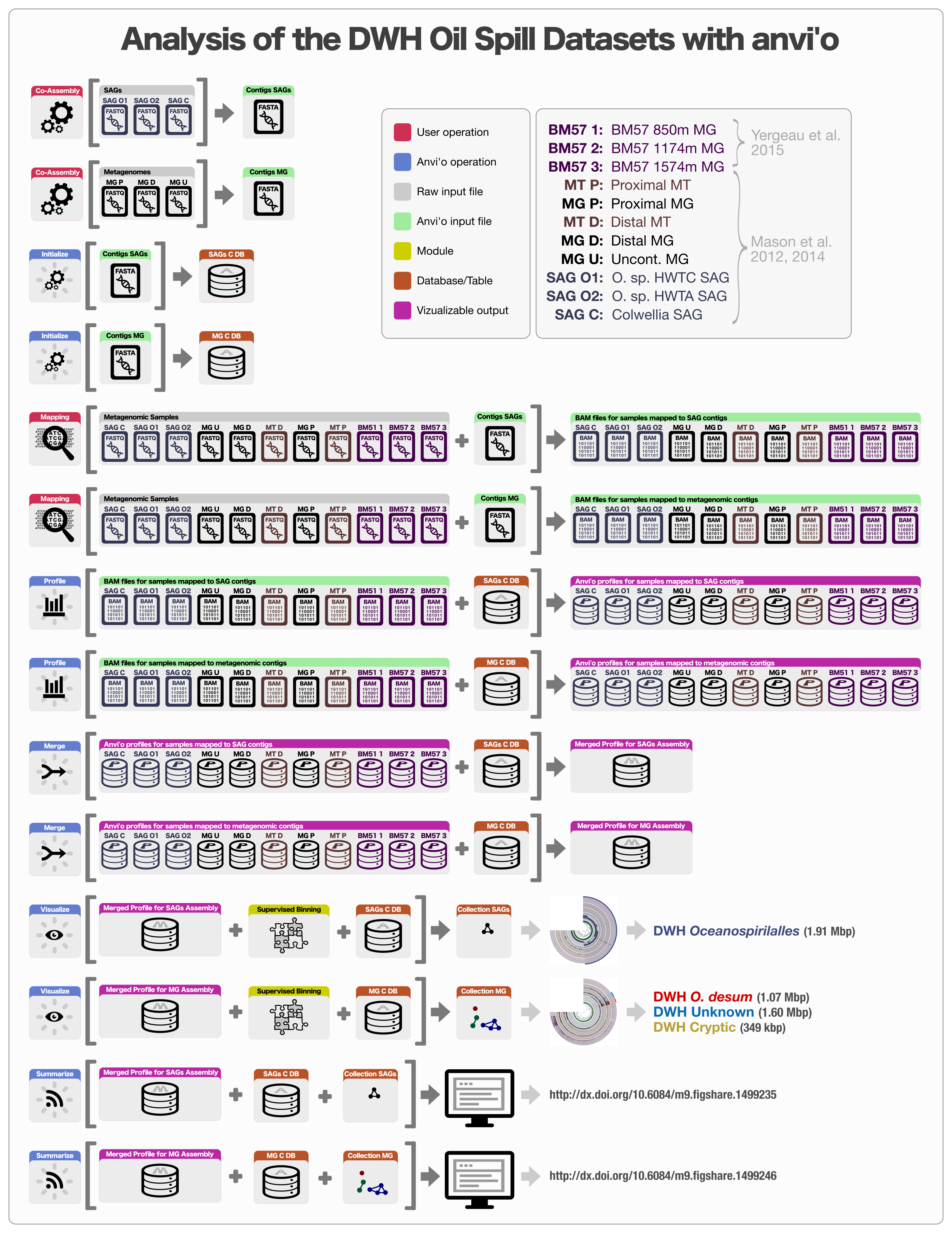

Supplement: Figure S1 [file peerj-03-1319-s004.png]

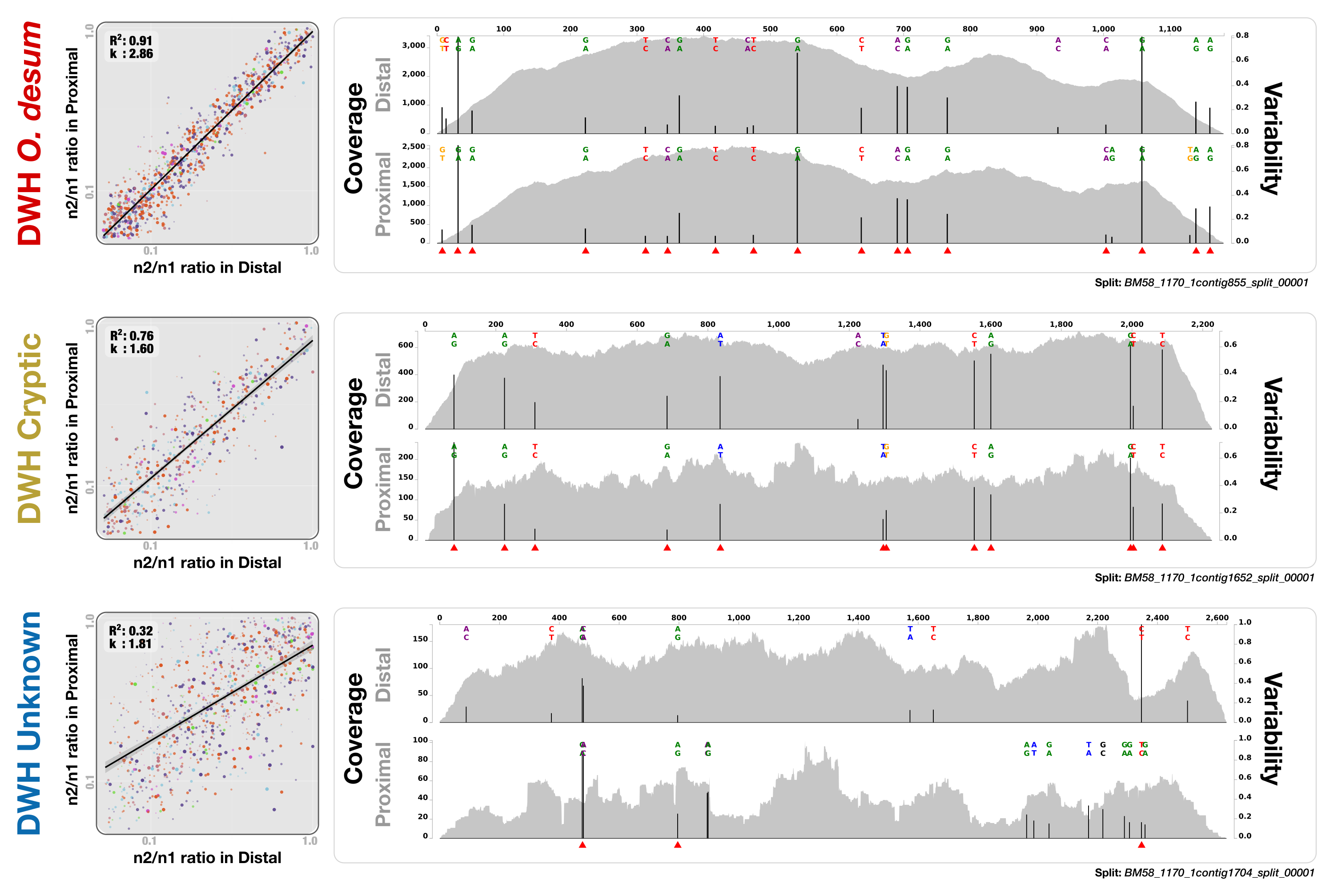

Supplement: Figure S2 — In each panel, plots on the left show the summary of all variable positions (see Fig. 5 and its caption for details) in a given genome bin, while each coverage/variability plot on the right demonstrates an example contig from a given genome bin. Red triangles underneath the variable nucleotide positions identify the positions that contribute to the generation of the plots on the left side. [file peerj-03-1319-s005.png]
